# Supplementary material for: Canine Gallbladder Erosion/Ulcer and Hemocholecyst: Clinicopathological Characteristics of 14 Cases
Source: Animals (Basel). 2023 Oct 26;13(21):3335. doi: 10.3390/ani13213335 (PMC10649012; doi:10.3390/ani13213335)
Supplement: Supplementary file 1 [file animals-13-03335-s001.zip › Supplemental material/Supplemental Figure S2.pdf]

Supplemental Figure S2

Gross findings of the affected gallbladders.
Blood coagula (arrowheads) are attached to the gallbladder mucosa.

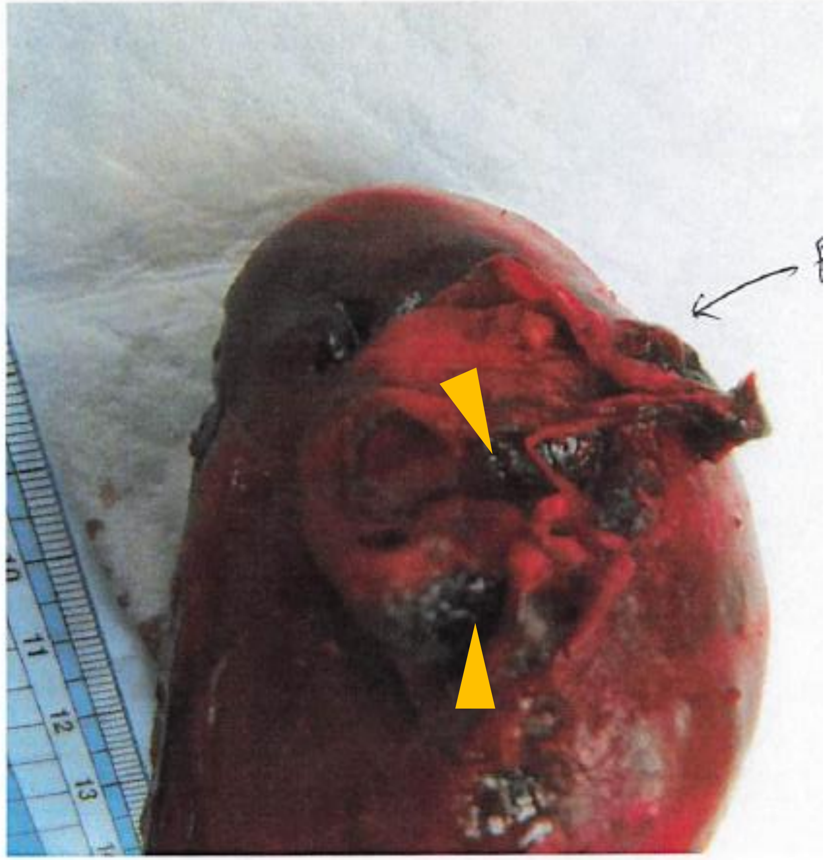

GU9 (formalin unfixed)

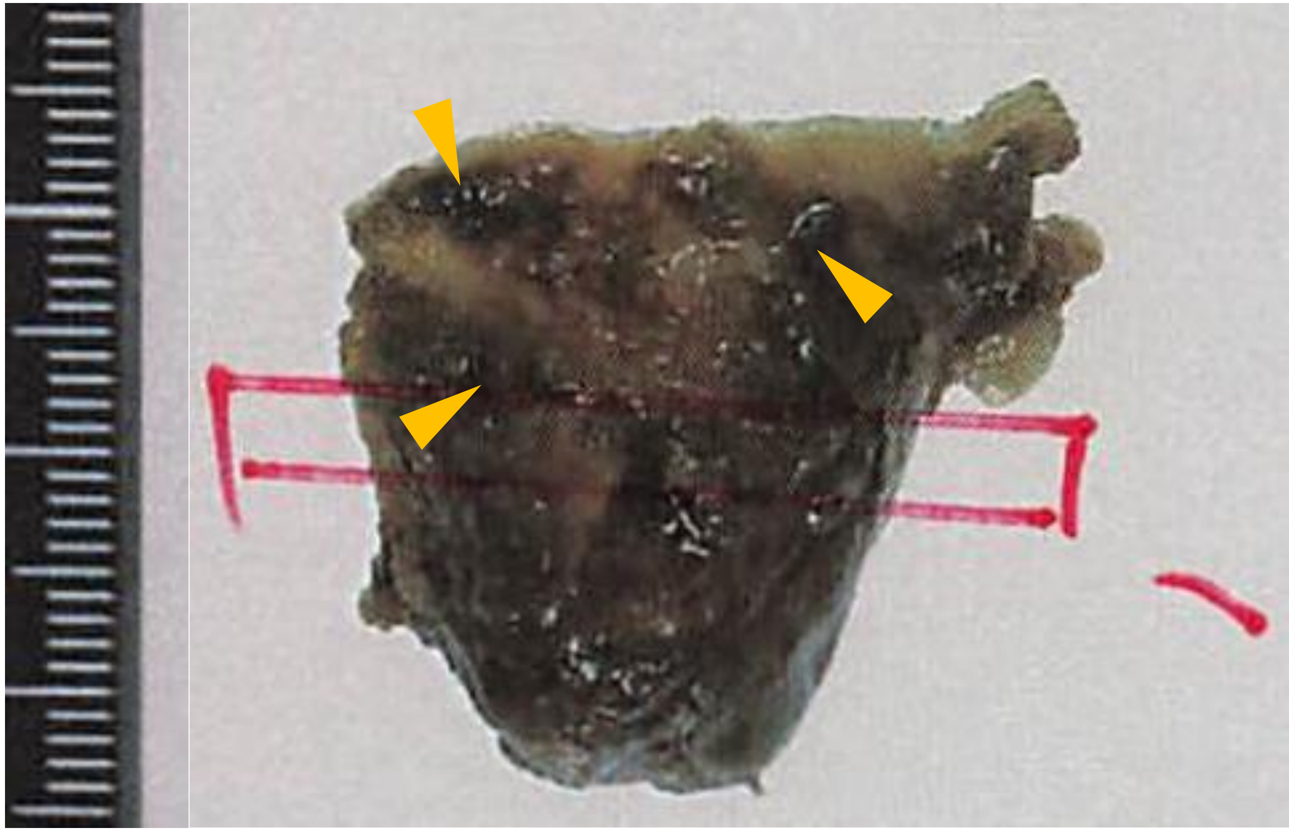

GU14 (formalin fixed)
