# Supplementary material for: Canine Gallbladder Erosion/Ulcer and Hemocholecyst: Clinicopathological Characteristics of 14 Cases
Source: Animals (Basel). 2023 Oct 26;13(21):3335. doi: 10.3390/ani13213335 (PMC10649012; doi:10.3390/ani13213335)
Supplement: Supplementary file 1 [file animals-13-03335-s001.zip › Supplemental material/Supplemental Table S1.pdf]

Supplemental Table S1

Signalment and results of clinicopathological investigation of 24 dogs.

Case number	Age[d]	Breed	Sex	Duration between clinical onset and cholecystectomy	Gallbladder rupture?	Chief complaint	Ultrasonographic findings of gallbladder	CBC[j]	Blood chemistry[k]
**GU[a]1**	103	Toy Poodle	M[e]	> 3 wks	Yes	Vomiting, lethargy	Mucocele	Leukocytosis with band neutrophils	TP[l] 4.0, Alb[m] 1.3, CRP[n] >7.0, TBA[o] 19.7 (pre)→33.2 (post), branched chain amino acids 187, thyroxine 16 2wk later TP 5.4 and Alb 1.8
**GU2**	162	Shetland Sheepdog	CM[f]	3 days	No	Anorexia, lethargy, vomiting, icterus	Distention, thickened wall	None	Elevated liver enzymes, T-Bil 6.3, ALP[p] >3500
**GU3**	170	Miniature Dachshund	CM	2 days	Yes	Lethargy	Gallbladder rupture	NA	Elevated liver enzymes
**GU4**	86	Mixed breed	SF[g]	2 wks	No	Anorexia, bilirubinuria, icterus	Material with shadowing	NA	ALP >2000, ALT[q] 869, AST[r] 163, GGT[s] 126, T-Bil[t] 5.2
**GU5**	157	Miniature Dachshund	SF	Same day	No	Anorexia, vomiting, diarrhea	Sludge, thickened wall	Anemia	Glu[u] 74, Alb 2.2, ALT 78, AST 421, ALP 650, CRP over
**GU6**	132	German Shepherd Dog	M	Same day	Yes	Vomiting, lethargy, ascites (yellowish sanguineous fluid)	NA	NA	NA
**GU7**	69	Mixed breed	CM	> 1 month	Yes	NA	NA	NA	NA
**GU8**	102	Yorkshire Terrier	F[h]	9 days	NA	Anorexia, lethargy, vomiting, icterus	Immovable contents	NA	ALT >1000, ALP >3500, Lip[v] >1000, T-Bil 1.0, elevation of T-Cho[w], GGT, CRP
**GU9**	120	Mixed breed	CM	Same day	NA	Collapse, hemoabdomen	NA	Mild anemia	Elevated ALT and ALP
**GU10**	166	Shiba Inu	CM	17 days	No	Anorexia, vomiting	Sludge, thickened wall	None	Elevation of T-Bil, liver enzymes, CRP
**GU11**	112	Miniature Dachshund	SF	Same day	No	Vomiting	NA	NA	Marked elevation of liver enzymes and T-Bil
**GU12**	127	Pembroke Welsh Corgi	SF	6 days	No	Anorexia, lethargy, vomiting	Mucocele, dilation of	NA	Marked elevation of T-Bil
**GU13**	104	Chihuahua	SF	Same day	Yes	Lethargy	Gallstone	Neutrophilia with left shift and toxic neutrophils	ALT 133, AST 55, ALP 3212, T-Cho 347, GGT 18
**GU14**	107	Mixed breed	CM	1 day	NA	Febrile	NA	None	ALT 248, AST 128, ALP 3083, T-Bil 0.7, T-Cho 438, GGT 16
**CC[b]1**	149	Miniature Dachshund	SF	No clinical sign	No	None	Fluid	None	No abnormality
**CC2**	30	French Bulldog	M	No clinical sign	No	Intermittent vomiting after meal, rare hematemesis	Sludge	NA	Elevated liver enzymes
**CC3**	161	Chihuahua	CM	NA[i]	No	NA	NA	NA	NA
**CC4**	146	Maltese	SF	No clinical sign	No	None	Sludge, gallstone	NA	NA
**CC5**	174	Miniature Dachshund	CM	3 days	No	Anorexia, icterus	Cholecystitis	Anemia	NA
**GM[c]1**	146	Border Collie	CM	No clinical sign	No	NA	Mucocele	NA	Elevated ALT and ALP
**GM2**	136	Shiba Inu	M	1 day	No	Anorexia, lethargy	Mucocele	Leukocytosis	Elevation of liver enzymes and T-Bil
**GM3**	150	Chihuahua	CM	1 day	No	Anorexia	Mucocele	NA	NA
**GM4**	126	Pomeranian	M	> 2 months	No	NA	Mucocele, marked distention, thickened wall	NA	NA
**GM5**	144	Miniature Schnauzer	CM	No clinical sign	No	NA	Mucocele	NA	Elevated liver enzymes

[a]gallbladder ulcer, [b]chronic cholecystitis, [c]gallbladder mucocele, [d]in months, [e]male, [f]castrated male, [g]spayed female, [h]female, [i]not available, [j]complete blood count, [j and k]no reference interval provided, [l]total protein, [m]albun
cholesterol, [x]present, [y]gallbladder wall total thickness in micrometer

Supplemental Table S1

Other findings provided by the submitters	Ultrasonographic sludge	Histologic mucocele	Histologic gallbladder contents	Histologic erosion and/or ulcer	Severe cholecystitis	Mild cholecystitis	No cholecystitis	GWTT[y]
Small intestinal lymphatic dilation upon laparotomy, gallbladder adhesion		P	Mucocele, blood, fibrin, bile	P			P	1760
Common bile duct occlusion			Mucus, bile, blood	P	P			1450
Ascites, recent rabies vaccination		P	Mucus, blood	P	P			3800
Opaque material on X-ray, **cholelith** in gallbladder and bile duct on CT, dilation and tortuosity of bile duct			Mucus, blood	P	P			1650
Splenic nodular masses (ultrasound, laparotomy: histologic diagnosis extramedullary hematopoiesis)	P[x]		Mucus, bile, fibrin, blood, suppurative exudate	P	P			1430
Ascites with cocci-laden neutrophils			Mucus, bile, blood	P	P			2130
Gallbladder adhesion		P	Blood	P			P (autolysis/necrosis)	1640
None			Blood, suppurative exudate	P		P		1300
Hepatic nodular mass (histologic diagnosis hepatocellular adenoma)			Blood	P		P		840
Common bile duct partial occlusion	P		Mucus, blood	P	P			1360
Microhepatia			Mucus, blood	P	P			1180
Dilation of bile duct			Mucus, blood	P	P			1700
Gallbladder suppurative exudate			Mucus, blood, suppurative exudate	P	P			1260
Peritonitis			Bile	P	P			2090
Fragile liver			None		P			912
Scant ascites upon laparotomy, pyloric stenosis palpated	P		None		P			1780
NA			None		P			1540
Bile duct dilation	P		None		P			1360
NA			None		P			1560
Small intestinal lymphangiectasis		P	Mucocele			P		1510
NA		P	Mucocele					1470
NA	P	P	Mucocele			P		1700
NA		P	Mucocele			P		2710
Diabetes mellitus (being treated with insulin), obese, hepatomegaly		P	Mucocele			P		481

nin, [n]C-reactive protein, [o]total bile acids, [p]alkaline phosphatase, [q]alanine aminotransferase, [r]aspartate aminotransferase, [s]γ-glutamyl transpeptidase, [t]total bilirubin, [u]glucose, [v]lipase, [w]total
